# Goniodysgenesis variability and activity of *CYP1B1* genotypes in primary congenital glaucoma

**DOI:** 10.1371/journal.pone.0176386

**Published:** 2017-04-27

**Authors:** María T. García-Antón, Juan J. Salazar, Rosa de Hoz, Blanca Rojas, Ana I. Ramírez, Alberto Triviño, José-Daniel Aroca-Aguilar, Julián García-Feijoo, Julio Escribano, José M. Ramírez

**Affiliations:** 1 Instituto de Investigaciones Oftalmológicas Ramón Castroviejo, Universidad Complutense de Madrid, Madrid, Spain; 2 Cooperative Research Network on Age-Related Ocular Pathology, Visual and Life Quality, Instituto de Salud Carlos III, Madrid, Spain; 3 Departamento de Oftalmología y ORL, Facultad de Óptica y Optometría, Universidad Complutense de Madrid, Madrid, Spain; 4 Departamento de Oftalmología y ORL, Facultad de Medicina, Universidad Complutense de Madrid, Madrid, Spain; 5 Área de Genética, Facultad de Medicina/Instituto de Investigación en Discapacidades Neurológicas (IDINE), Universidad de Castilla-La Mancha, Albacete, Spain; 6 Servicio de Oftalmología, Hospital Clínico San Carlos, Instituto de Investigación Sanitaria del Hospital Clínico San Carlos, Madrid, Spain; University of Iowa, UNITED STATES

## Abstract

Mutations in the *CYP1B1* gene are currently the main known genetic cause of primary congenital glaucoma (PCG), a leading cause of blindness in children. Here, we analyze for the first time the *CYP1B1* genotype activity and the microscopic and clinical phenotypes in human PCG. Surgical pieces from trabeculectomy from patients with PCG (n = 5) and sclerocorneal rims (n = 3) from cadaver donors were processed for transmission electron microscopy. Patients were classified into three groups depending on goniodysgenesis severity, which was influenced by CYP1B1 enzymatic activity. The main histological changes observed in the outflow pathway of patients with PCG and mutations in *CYP1B1* were: i) underdeveloped collector channels and the Schlemm’s canal; ii) abnormal insertion of the ciliary muscle; iii) death of the trabecular endothelial cells. Our findings could be useful in improving treatment strategy of PCG associated with *CYP1B1* mutations.

## Introduction

Primary congenital glaucoma (PCG) is a major cause of blindness in children. It is usually diagnosed before the age of 3 years (neonates or infants) and it is transmitted as an autosomal-recessive trait with incomplete penetrance [1, 2]. Clinical manifestation includes buphthalmos as a result of high intraocular pressure (IOP), edema and opacification of the cornea with rupture of Descemet’s membrane, photophobia, and excessive tearing, among other symptoms. PCG results from developmental defects of the trabecular meshwork (TM) (trabeculodysgenesis) which is responsible for increased aqueous outflow resistance, elevated IOP, and optic nerve damage [3].

The level of incidence varies across different ethnic groups. A high incidence has been found among Slovakian gypsies (1/1250) [4] and Saudi Arabian (1/2500) [2] populations, but lower rates in Western countries (1/5000-1/10000) [5].

Mutations in the *CYP1B1* gene are currently the main known genetic cause of PCG in Spanish patients [6] and in different worldwide populations [7–14].

*CYP1B1* is a member of the cytochrome P450 superfamily that catalyzes NADPH-dependent monooxygenation of diverse xenobiotics and endogenous molecules [15, 16]. It has been demonstrated that glaucoma-associated *CYP1B1* mutations reduce the enzymatic activity or stability of the enzyme and diminish the localization of the protein in the mitochondria [17–19]. C*YP1B1* expression is conserved in early embryos across several species during development of the ocular tissues [20]. It is speculated that this enzyme participates in the normal development of the TM tissue [21]. It has been shown that approximately 30% of Spanish PCG patients carry loss-of-function *CYP1B1* variants, with most of these variants, resulting in null genotypes [6]. Incomplete penetrance, variable expressivity, and the finding of a significant proportion of patients who carry non-dominant heterozygous *CYP1B1* mutations [6] suggest that more than one gene can be involved in PCG inheritance.

Studies examining the correlation between the histological alterations in the aqueous outflow pathways in congenital glaucoma and the different mutations in the *CYP1B1* gene are scarce and difficult to conduct. Only 4 cases are available in the literature [22] in which the histological changes in specific *CYP1B1* mutations associated with severe- or moderate-angle abnormalities are described. These genotype-phenotype correlations could be of clinical interest in PCG, and may help to provide more accurate prognosis, guide therapy, and assist in genetic counseling. This study seeks to increase the casuistry of studies analyzing the correlation between: the *CYP1B1* mutations, the histological changes found in the aqueous outflow pathway, and the severity of the clinical observations in patients with PCG.

## Materials and methods

The PCG patients included in the present study were recruited from among the outpatients of the Ophthalmology Department of the Hospital Clínico San Carlos de Madrid (HCSC) (Madrid, Spain). All the patients suspected of having PCG were subjected to a full examination by an ophthalmologist specializing in congenital glaucoma including: slit-lamp biomicroscopy, gonioscopy, IOP, pachymetry, corneal diameter, biometry and ophthalmoscopy. In most cases, examination under general anesthesia was necessary both for diagnosis and follow-up purposes. IOP was measured as soon as the child was sufficiently anesthetized (during the first 10 min after induction) and again immediately before the child fully regains consciousness. In these instances, the IOP was checked using the Perkins applanation tonometer (Clement Clarke MK2 applanation tonometer, Clement Clarke International Ltd, Harlow, UK).

The diagnosis of PCG was based on the presence of at least 2 of the following clinical features: i) increased corneal diameter (>12mm) together with elevated IOP (>21mm Hg or >16mm Hg under general anesthesia); ii) Haab’s striae, iii) corneal edema, and iv) optic-disc changes. The patients described in this study required trabeculectomy in at least one eye for IOP control.

For the *CYP1B1* gene analysis peripheral blood samples were collected from each patient.

Five patients met the criteria to be included in the study; all of them had been diagnosed with PCG and were carriers of a *CYP1B1* gene mutation. No additional patients were enrolled and later excluded from the study.

Written informed consent for tissue study, analysis, and publication of the results were given by the next of kin of each subject included in the study.

In addition, three adult human normal sclerocorneal discs from cadaver donors (45, 73, and 76 years old) were used as control of normal ultrastructure of the trabecular meshwork (TM), the juxtacanalicular tissue (JCT) and the Schlemm’s canal (SC), in order to facilitate the interpretation of the morphological changes observed in the specimens from patients with congenital glaucoma.

The study was approved by our institutional ethics committee (CEIC Hospital Clínico San Carlos), and the work was conducted within the tenets of the Declaration of Helsinki.

### Mutation analysis

The *CYP1B1* gene study was performed at the Human Molecular Genetics laboratory of the Faculty of Medicine, Universidad de Castilla-La Mancha (Campus de Albacete, Spain). Genomic DNA was extracted from the peripheral leukocytes of the five subjects studied using the QIAamp DNA Blood Mini Kit (Qiagen, Hilden, Germany) according to the manufacturer’s protocol. The coding (exons II and III), untranslated (exon I), and promoter (nucleotides −1 to −867) regions of *CYP1B1* were amplified as previously described [23]. Terminator cycle sequencing was performed using the BigDye (v3.1) kit (Applied Biosystems, Foster City, CA), and the products of sequencing reactions were analyzed in an automated capillary DNA sequencer (ABI Prism 3130 genetic analyzer; Applied Biosystems). Mutations were confirmed by independent sequencing in a second DNA sample [19].

### Tissue processing

Standard trabeculectomies were performed in all patients under general anesthesia in the HCSC (Madrid, Spain). A limbus-based conjunctival flap was raised, and a half-thickness scleral flap was dissected. The tissue at the anterior-chamber angle was dissected. The trabeculectomy tissues and the sclerocorneal disc from donors were fixed immediately by immersion in 2% glutaraldehyde in 0.1 M phosphate buffer (PB), pH 7.4 for 5 h at 4°C. Then the tissues were brought to Instituto de Investigaciones Oftalmológicas Ramón Castroviejo at the Universidad Complutense de Madrid (Spain) for their subsequent histological processing. After fixation, the tissues were washed in PB (pH 7.4) and then post-fixed in 1% osmium tetraoxide for 2h at 4°C. The tissues were then dehydrated in ascending grades of acetone series (30–100%) and embedded in araldite. The semi-thin sections (0.5 μm) were stained with toluidine blue, and after selection, the blocks were further trimmed for ultramicrotomy (Reichert OM-V3 ultramicrotome, Wetzlar, Germany). The thin sections were contrasted with uranyl acetate and lead citrate, and examined and photographed by transmission-electron microscopy (TEM; JEOL JEM 1010). The tissue-sample study was performed by a PhD in neurobiology and MD PhD in ophthalmology with long experience in the ocular histopathology.

Given that the criteria to be included in the present study was to have PCG and *CYP1B1* gene mutation, the scientists performing the ultra-structural analysis were aware of this data, although any information regarding the patient who corresponded to the surgical sample analyzed or the enzymatic activity associated with each specific genotype were masked for the observers.

## Results

Five patients (four females and one male) met the criteria to be included in the study. Each of these patients were diagnosed with bilateral PCG and open angles upon examination at HCSC. Mean follow-up for patients ranged from 3 to 10 years. During the postoperative period, the target IOP control was achieved in all cases with both surgical procedures and topical glaucoma medications. *CYP1B1* mutations were present in the five patients (100%). The mutations identified in the probands and some of their family members are listed in “Table 1”.

**Table 1:** *CYP1B1* mutations in primary congenital glaucoma (GCP).

Case (Family)	Ethnicity	*CYP1B1* genotype	Genotype activity* (% of wild type)
1 (PCG-151)	Arab	p.Ala179ArgfsTer18/p.Ala179ArgfsTer18	0
2 (PCG-49; II:4)	Caucasian	p.Arg355HisfsTer69/p.Thr404SerfsTer30	00
3 (PCG-49; II:2)	Caucasian	p.Arg355HisfsTer69/p.Thr404SerfsTer30	00
4 (PCG-69)	Caucasian	p.Glu387Lys/p.Glu387Lys	00
5 (PCG-207)	Hispanic	p.Gly61Glu/+	60

* Genotype activity was estimated assuming a biallelic *CYP1B1* gene-expression model in which the enzymatic activity associated with each genotype was the mean of the sum of the activity of the two individual alleles.

p.Ala179ArgfsTer18: c.535delG, rs771076928.

p.Arg355HisfsTer69: c.1064_1076delGAGTGCA, rs72549380.

p.Glu387Lys: c.1159G>A, rs55989760.

p.Gly61Glu: c.182G>A, rs28936700.

p.Thr404SerfsTer30: c.1200_1209dupTCATGCCAC, rs587778873.

### *CYP1B1* genotypes and associated enzymatic activity

**Fig 1:**
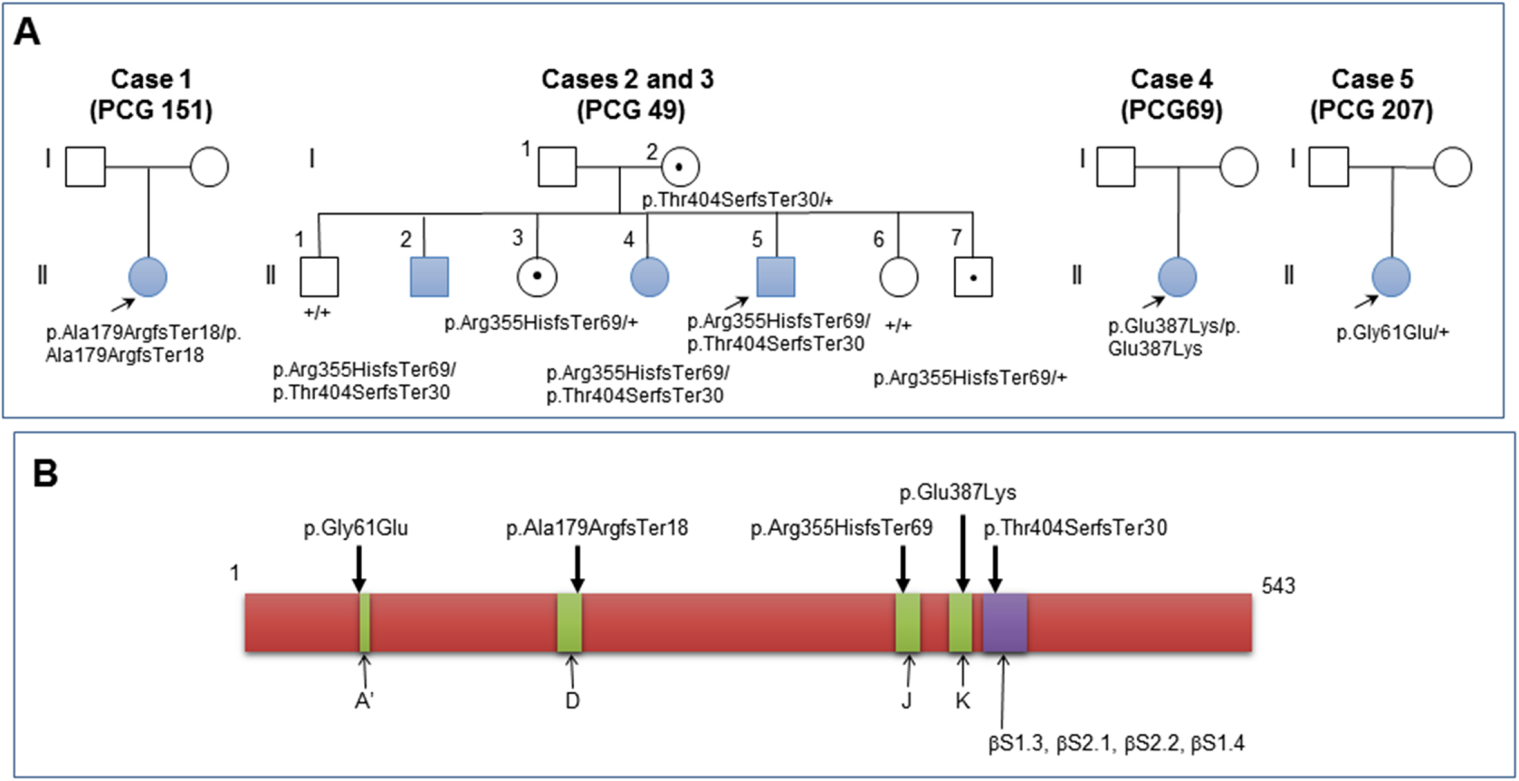
**A: Pedigrees of congenital glaucoma families included in this study.** *CYP1B1* genotypes are indicated below the symbols. Arrows show probands. Blue symbols indicate glaucoma phenotypes. Carriers are denoted by black dots in symbols; +: wild-type allele. Genealogy PCG 49 has previously been reported [6, 24] **B: Location in the polypeptide chain of the *CYP1B1* mutations identified in the probands**. Alpha-helix regions are indicated in green and indicated with capital letters (A’-K). βS: beta-sheet regions (blue box). The numbers above the scheme correspond to amino acid positions.

The five cases selected for this study were carriers of different combinations of five different *CYP1B1* sequence variants “Table 1”. Genotypes of patients PCG 49 and PCG 69 have previously been reported [6, 24]. Three patients had no brother or sister and were the only affected members of their families “Fig 1A”. Two of the patients were siblings (PCG 49, II:2 and II:4) and belonged to an seven member family with three affected subjects who carried the same genotype but manifested the disease at different ages “Fig 1A”, clearly illustrating the clinical variability present in *CYP1B1*-associated glaucoma [24]. Three out of the five cases were compound heterozygotes, one was homozygote, and the last one was a single heterozygote. The five variants affected important structural elements of the polypeptide chain “Fig 1B”. Previous functional studies have shown that one of the variants (p.Gly61Glu) was hypomorphic (20% enzymatic activity) and the remaining four variants were null alleles (0% enzymatic activity) [6, 19]. These data led us to infer that the enzymatic activity associated with four of the patient genotypes was null while it was approximately 60% of the wild-type genotype in patient PCG 207 “Table 1”.

### Phenotype expression

**Fig 2:**
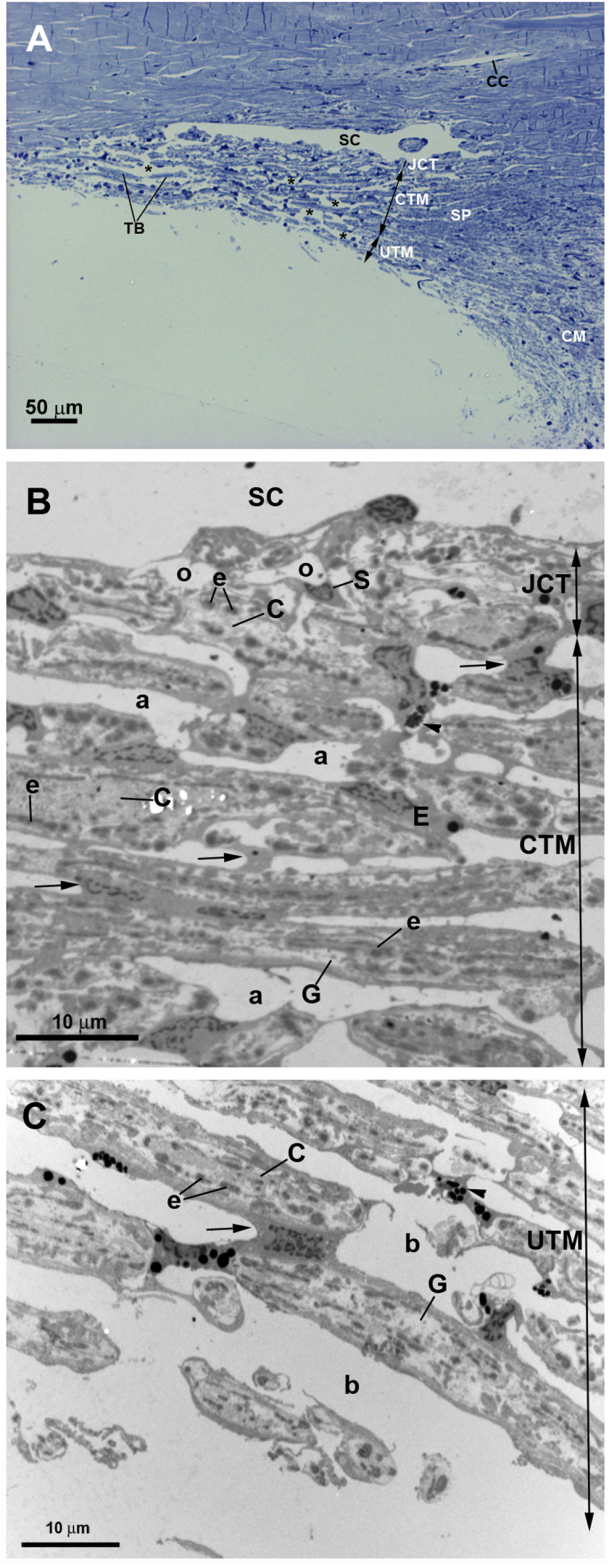
Anterior-chamber angle of normal eyes. A: Light micrograph. B and C: Transmission electron microscopy. B: Juxtacanalicular tissue (JCT) and corneoscleral trabecular meshwork (CTM). C: Uveal trabecular meshwork (UTM). A: The ciliary muscle (CM) is inserted backwards to the scleral spur (SP). Schlemm’s canal (SC) and the collector channel (CC) have an open lumen. The trabecular meshwork is constituted by well-developed trabecular beams (TB) and intertrabecular spaces (*). Two regions can be distinguished in the TM: the CTM, formed by 6 to 8 layers of trabecular beams and the UTM composed of 2 to 3 layers of trabecular beams. B and C: The JCT (in B) is composed by stellate cells (S) and a loose extracellular matrix in which collagen (C), elastic fibers (e), and numerous “optically empty spaces” (o) are visible. The trabecular beams both in CTM and UTM (in B and C) are constituted by a central core made up mainly by collagen (C), elastic-like fibers (e) and amorphous ground substance (G). This central core is coated by phagocytic endothelial cells (E) that can bridge the intertrabecular spaces (arrow). The main difference between CTM (in B) and UTM (in C) is the size of the intertrabecular spaces, being larger in UTM (b) than in CTM (a). [arrowhead: pigmented granules phagocyted].

In control eyes the ciliary muscle was inserted backwards to the scleral spur “Fig 2A”. The SC had an open lumen “Fig 2A and 2B”. The JCT lies adjacent to the inner wall of the SC and was composed by stellate cells immersed in a loose web of extracellular matrix (ECM) in which collagen fibers, elastic fibers and numerous empty spaces were identified “Fig 2B”. The TM consisted of a well-developed meshwork constituted by trabecular beams and intertrabecular spaces “Fig 2A–2C”. The TM was divided into two regions: the corneoscleral) trabecular meshwork (CTM) and the uveal trabecular meshwork (UTM) “Fig 2A”. The CTM extended from the scleral spur to the Schwalbe’s line and was composed by six to eight layers of trabecular beams “Fig 2A”. The UTM was closer to the anterior chamber than CTM; arose from the uveal tract (root of the iris and ciliary body) and was constituted by two to three layers of trabecular beams “Fig 2A”. In the UTM the intertrabecular spaces were substantially larger “Fig 2C” than in CTM, the latter being more elliptical in shape “Fig 2B”. The trabecular beams comprised a central core of connective tissue composed mainly of collagen and elastic fibers and amorphous ground substance. This central core was coated by trabecular endothelial cells that could bridge the intertrabecular spaces, thus covering two adjacent trabecular beams “Fig 2B and 2C”. In addition, phagocyted pigment granules were detected in some trabecular endothelial cells, indicating high phagocytic activity “Fig 2B and 2C”.

The histological analysis of the 5 cases of PCG with *CYP1B1* mutations included in the present study revealed that the degree of developmental anomalies of the aqueous pathways varied among patients. We classified the patients into three groups according to the goniodysgenesis severity. Group A: no visualization of the SC and the collector channels; group B: presence of the SC and no visualization of the collector channels and; group C: presence of the SC and the collector channels.

### Group A

#### Case 1

This case corresponded to a 7-year-old Arab girl who was diagnosed with PCG at the age of 4 months. When first seen at HCSC (21-months-old) she had undergone two goniotomy and one trabeculectomy in both eyes (OU). The ophthalmological examination revealed an increased IOP (35 mmHg) and an enlargement of the corneal diameter OU despite being on topical treatment with two glaucoma drugs. A third goniotomy and a second trabeculectomy were then performed OU. At the age of 3, the IOP rose again so that a new trabeculectomy (the 3rd) OU was undertaken. During the following years, topical glaucoma treatment with three drugs was necessary to keep the IOP under control. At the age of 5, an Ahmed valve was implanted due to bilateral corneal edema which resulted in recovery of the corneal transparency and control of the IOP. Since then, the target IOP has been achieved without topical glaucoma medication in the right eye (OD) and with one glaucoma drug in the left eye (OS) for up to 2 and a half years of follow-up “Table 2”.

**Table 2:** Correlation of slit–lamp findings, histological-angle anomalies, and treatment in five patients with PCG and *CYP1B1* mutations.

Case (Family)	Sex	Age at diagnosis	Slit–Lamp findings	Histological anomalies	Goniodysgenesis severity	Treatments						Follow-up
						Goniotomy	Trabecu-lotomy	Trabecu-lectomy	Ahmed valve	Drugs	No of Surgeries	
1 (PCG-151)	F	4 months	- Corneal enlargement - Edema OU	- SC and CC not observed - Abnormal CM insertion - TB fused - CF increased - TEC necrosis	+++	3 OU		3 OU	1 OU		7 OU	7y
2 (PCG-49; II:4)	F	At birth	- Catarata OU - Peripheral leukoma OU - Haab striae OU	- CC not observed - Abnormal CM insertion - TB fused - CF increase - TEC necrosis	++		2 OU	1 OD 2 OS	1 OU pediatric 1 OS adult	1 OU	3 OD 6 OS	10y
3 (PCG-49; II:2)	M	5 years	- Corneal enlargement OU	- CC not observed - In CTM TB fused - GS increase - TEC necrosis	++	1 OS		1 OS		3OD 4OS	2 OS	8y
4 (PCG-69)	F	At birth	- Corneal enlargement - Edema OU - Haab striae OU	- CC not observed - Abnormal CM insertion- - In CTM TB partially fused - CF increased - TEC necrosis	++	3 OU		1 OU		3 OS	4 OU	7.8y
5 (PCG-207)	F	1 month	- Corneal enlargement - Edema OU - Haab striae OD - Leukoma OS	- Abnormal CM insertion - Necrosis TEC only in UTM - CF increased	+			1 OU			1 OU	3y

CC: collector channels; F: female; M: male; OU: both eyes; OD: right eye; OS: left eye; SC: Schlemm’s Canal; TM: trabecular meshwork; CTM: corneoscleral trabecular meshwork; UTM: uveal trabecular meshwork; CM: ciliary muscle; TB: trabecular beams; CF: collagen fibers; TEC: trabecular endothelial cells; GS: ground substance.

**Fig 3:**
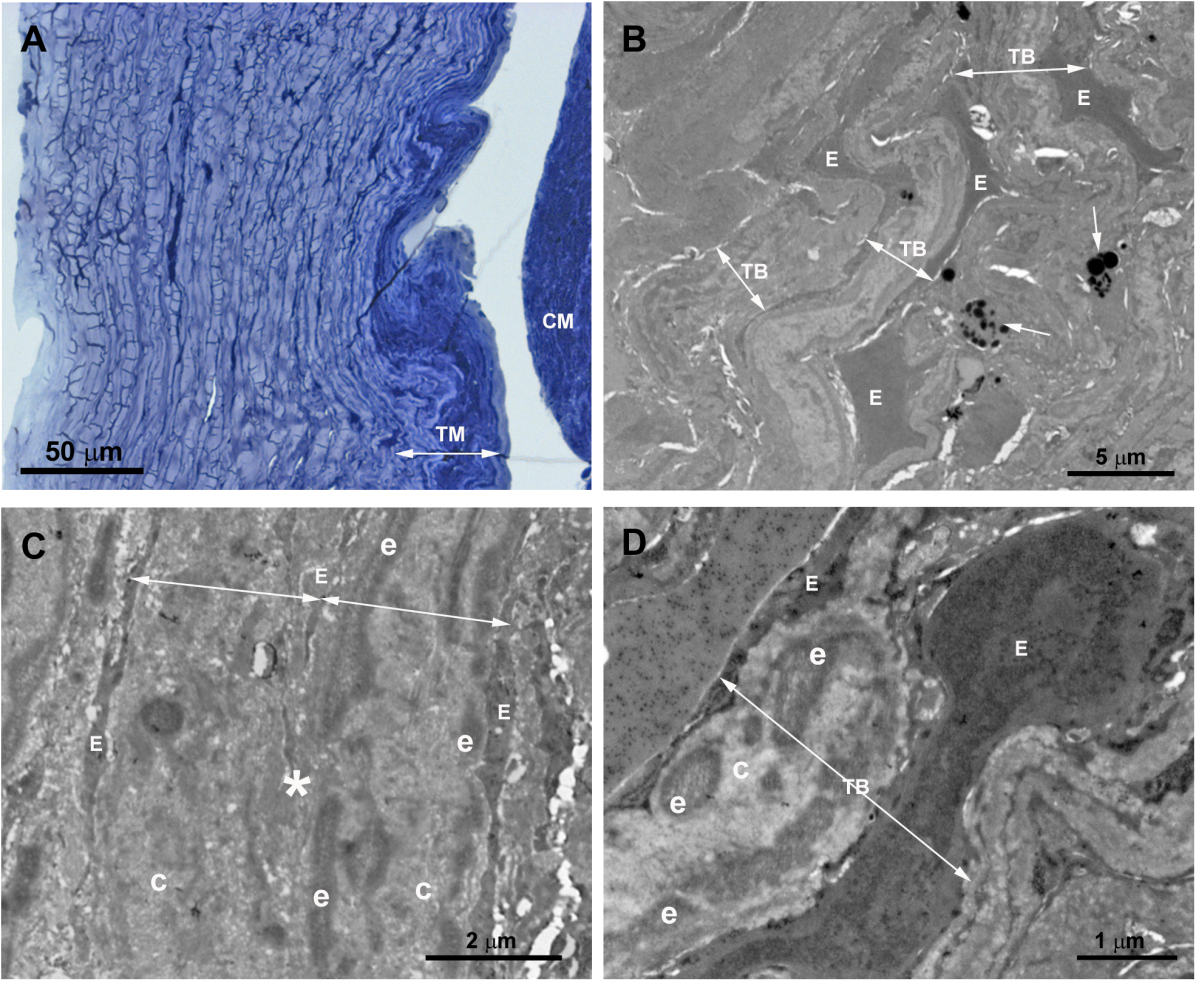
Case 1. **Trabeculectomy sample from the right eye.** A: Light micrograph. B-D: Transmission electron microscopy. A: Schlemm’s canal is absent. The region corresponding to the trabecular meshwork (TM) consists of a compact tissue. The ciliary muscle (CM) is located in front of the undifferentiated TM. B: Compacted trabecular beams (TB) (double arrows). Large endothelial cells (E) lining some of the trabeculae. Melanin granules (arrow). C: The image shows two trabecular beams fused (double arrows), the endothelial coating has disappeared at the point of fusion (asterisk). D: The trabecular core is filled by collagen (C) and elastic-like tissue (e). An enlarged endothelial cell (E) between two trabecular beams (TB) is shown. [double arrow: trabecular beam].

The tissue analyzed in this patient was obtained from the third trabeculectomy in the OD. In semithin sections, we observed that the area corresponding to the outflow pathway was replaced by a compact tissue in which neither the SC nor other components of the aqueous outflow pathway could be identified “Fig 3A”. At TEM, the region corresponding to the UTM and CTM comprises a compact tissue constituted by trabecular beams with no intertrabecular spaces “Fig 3B”. At higher magnification, it could be seen that in some areas, the trabecular beams fused together due to endothelial cells loss “Fig 3C”. The trabecular beams were constituted by collagen, elastic-like fibers and large endothelial cells “Fig 3B–3D”, some of them being necrotic. The collagen, predominantly of fibrillary type, was the main component of the trabecular beam “Fig 3C and 3D”. The ciliary muscle was abnormally inserted, overlapping the undifferentiated trabecular meshwork “Fig 3A”.

### Group B

#### Case 2

This case corresponds to a 12-year-old Caucasian girl who was diagnosed at birth of bilateral PCG. At the age of 4, she presented the HCSC with a past medical history of two trabeculotomies (at 7 and 15 days old) and a pediatric Ahmed valve (at 8 months old) OU. At age 6, she underwent a trabeculectomy in the OS due to poor control of IOP despite being on topical glaucoma treatment with 3 drugs. During the following 3 years, the IOP in the OS was pharmacologically controlled. At age 9, she required a trabeculectomy OU. Five months later, the pediatric Ahmed valve was replaced by an adult implant in OU. Since then, the IOP has been controlled OU for up to 3 years of follow-up with one topical glaucoma medication “Table 2”.

**Fig 4:**
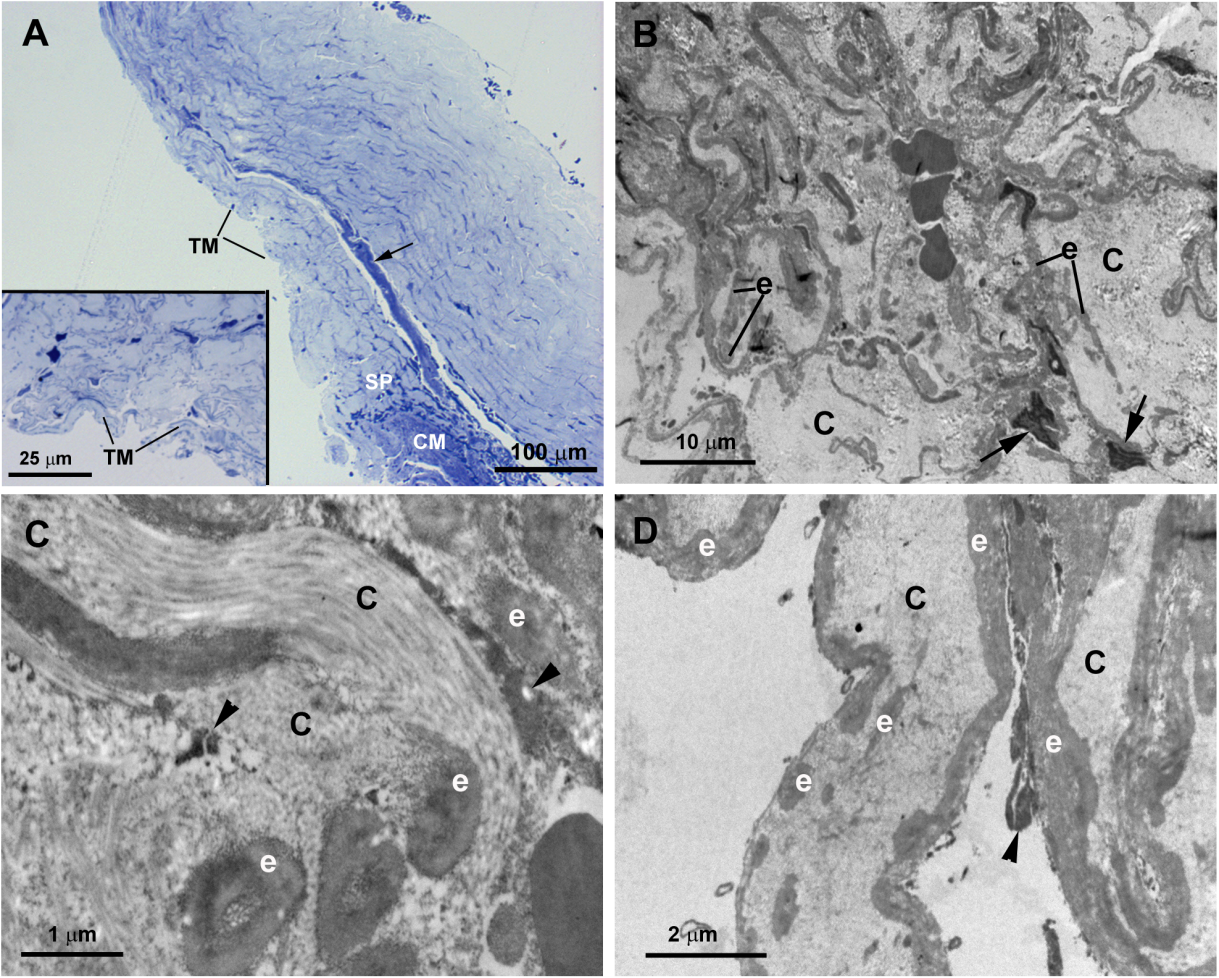
Case 2. **Trabeculectomy sample from the right eye**. A: Light micrographs. B-D: Transmission electron microscopy. A: Some remains of trabecular meshwork (TM) and an anterior ciliary vessel (arrow) are observed. No Schlemm’s canal or collector channels are visible. The ciliary muscle (CM) is inserted posterior to the trabecular meshwork (TM). Inset in A: Higher magnification of TM. B-D: In the area corresponding to the trabecular beams a compact tissue composed of abundant coalescent fibrillary collagen (C) and large amount of elastic-like fibers (e) is observed. The scarce endothelial cells lining the trabeculae show features of necrosis (arrowhead in C and D) and apoptosis (arrow in B). [SP: scleral spur].

The tissue analyzed in this instance was taken from the trabeculectomy of the OD. The histology of this surgical sample was influenced by the previous trabeculotomies suffered by the patient. For this reason, the SC and JCT could not be appropriately evaluated. However, some rest of trabecular beams, as observed in the semithin section “Fig 4A”, remained, allowing its analysis. No collector channels were detected “Fig 4A”. The analysis of the ultrastructure revealed that in the region corresponding to the TM remains there was a compact tissue composed of abundant coalescent fibrillary collagen, large amount of elastic fibers and scarce endothelial cells which showed signs of necrosis and apoptosis “Fig 4B”. It was difficult to identify the trabecular beams and no intertrabecular spaces were observed “Fig 4B and 4C”. By contrast, in some areas the trabecular beams and the intertrabecular spaces were present “Fig 4D”. Most endothelial cells lining these trabecular beams had disappeared, those remaining showing signs of necrosis and apoptosis “Fig 4D”. The trabecular beams were filled by coalescent fibrillary collagen and abundant elastic-like fibers “Fig 4C and 4D”. The ciliary muscle was inserted posterior to the TM “Fig 4A”.

#### Case 3

This case corresponds to a 16-year-old Caucasian male, who was diagnosed with PGC at the age of 5 and was brother of case 2. He was on medical treatment (one drug) for elevated IOP from age 5. At presentation in HCSC, an enlarged corneal diameter was the only sign detected. At the age of 16 a goniotomy in the OS was performed because of poor IOP control despite being on three topical glaucoma medications. Five months later, a trabeculectomy was required in the OS, achieving IOP control since then without medication. The IOP in the OD has been controlled up to the present with three topical glaucoma medications, and no previous surgical procedures were required.

**Fig 5:**
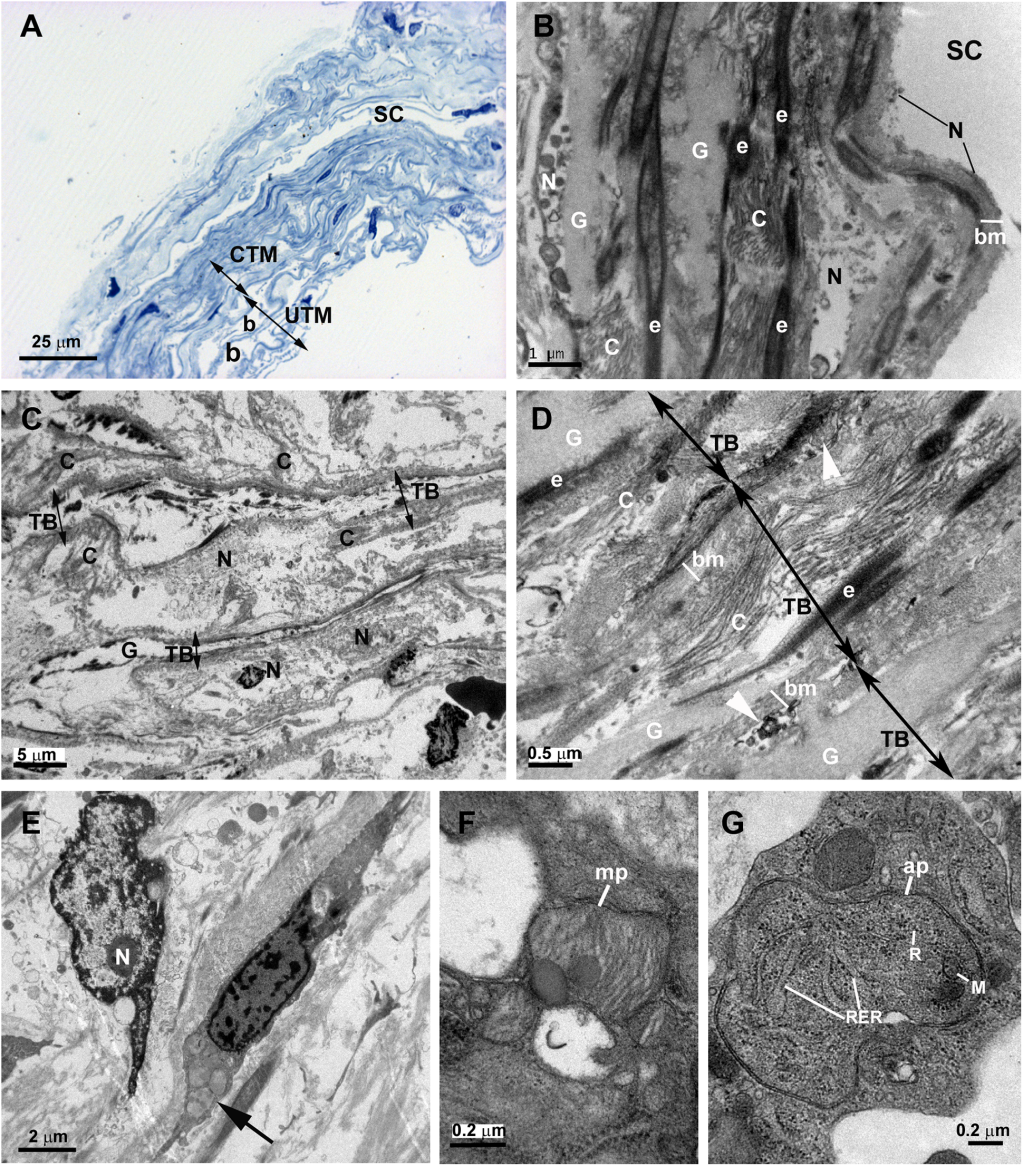
Case 3. **Trabeculectomy sample from the left eye.** A: Light micrograph. B-G: Transmission electron microscopy. A: Schlemm’s canal (SC) has an open lumen. The corneoscleral trabecular meshwork (CTM) is constituted by quite compact trabecular beams. In the uveal trabecular meshwork (UTM) the intertrabecular spaces (b) are evident. B: The image shows a part of Schlemm’s canal (SC) and the juxtacanalicular tissue. In the SC, only debris of necrotic endothelial cells (N) is visible on a thick basal membrane (bm). In the juxtacanalicular tissue, alternate layers of elastic tissue (e), necrotic cells (N), fibrillary collagen (C) and abundant ground substance (G) are seen. C: The corneoscleral trabecular meshwork is constituted by a quite disintegrated tissue. The trabecular beams (TB) (double arrows) have an “empty” appearance and are separated by intertrabecular spaces filled with debris of necrotic endothelial cells (N) and detached collagen (C) from the trabecular beams. D: The image shows fused trabecular beams (TB) (double arrows) and in some zones between them, debris from necrotic cells (white arrowhead) appears. The trabecular beams is constituted by a basal membrane (bm), many elastic-like fibers (e), fibrillary collagen (C), and abundant ground substance (G). E: The image shows two endothelial cells, one shows necrosis (N) and the other one has autophagic activity (arrow). F and G: Detail of the autophagic activity of an endothelial trabecular cell. F: Mitophagy (mp). G: Autophagosome (ap) with a double-membrane containing cytoplasmic material and organelles: mitochondria (M), rough endoplasmic reticulum (RER) and ribosomes (R).

The trabeculectomy from the OS of this patient contained two pieces of tissue, one corresponding to a part of the sclera and another containing the rest of the sclera, the SC and the TM. In semithin sections, we observed a SC with an open lumen, a quite compact CTM, and an UTM with well-developed intertrabecular spaces “Fig 5A”. In either pieces of sclera, we found no collector channels. The TEM revealed that most of the SC endothelial cells had disappeared with only debris of necrotic cells being observed “Fig 5B”. The JCT was composed of alternating layers of abundant ground substance, elastic-like fibers, fibrillary collagen and necrotic stellate cells “Fig 5B”. The CTM was difficult to recognize because the tissue was quite disorganized “Fig 5C”. In some areas the trabecular beams were separate and the zones that would correspond with the inter-trabecular spaces were occupied by the remains of necrotic cells and by collagen that had been detached from the trabeculae “Fig 5C”. In other areas the trabecular beams were fused, the boundary between them being recognized by the presence of necrotic cells debris “Fig 5D”. The trabecular beam had basal membranes, many elastic-like fibers, fibrillary collagen and abundant ground substance “Fig 5D”; the latter gave to the trabecular beam an “empty” appearance “Fig 5C”. The endothelial cells that delineate the trabecular beams were mostly necrotic “Fig 5E”, and those remaining showed autophagic activity “Fig 5E–5G”.

**Fig 6:**
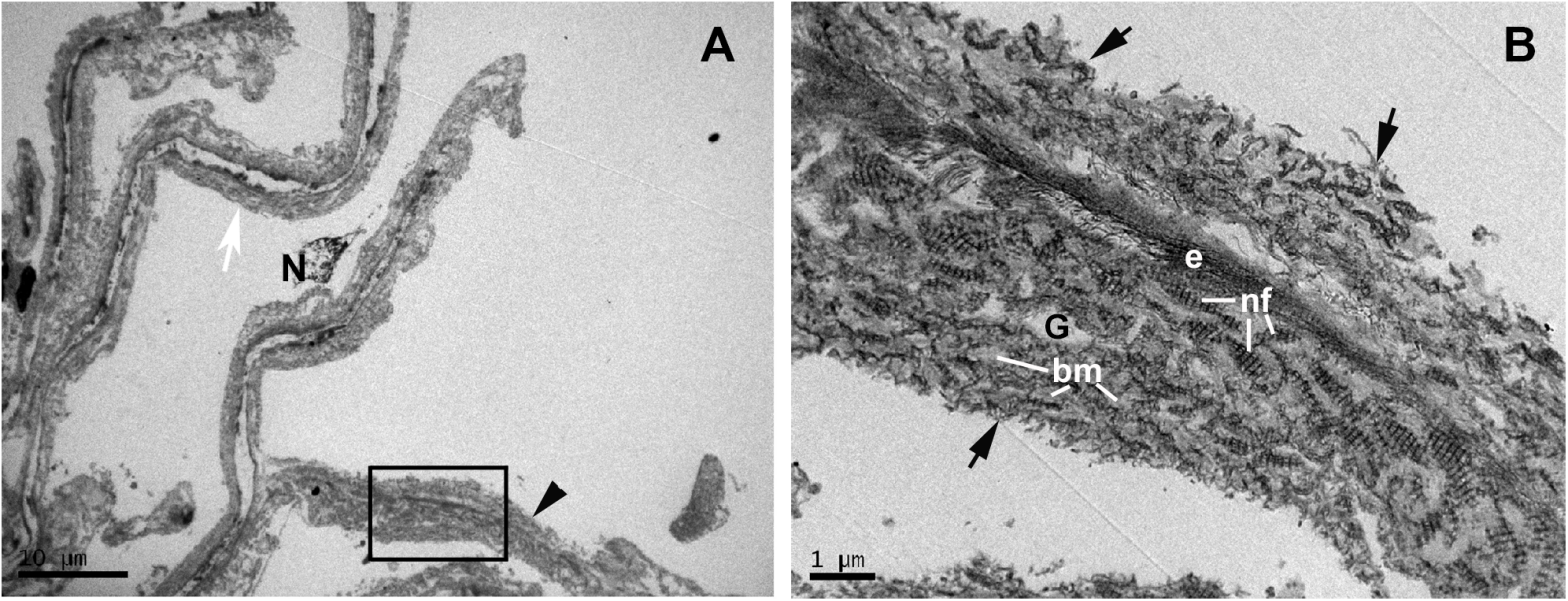
Case 3. **Trabeculectomy sample from the left eye.** Transmission electron microscopy. A: Uveal trabecular meshwork. B: High magnification of inset in A. The endothelial cells of the trabecular beams have disappeared and the remains are necrotic (N). In some areas, the trabecular beams have an empty appearance (white arrow) while others are completely filled (arrowhead) with type VI non-fibrillary collagen (nf) intermingled with the collagen of the basal membrane (bm), as observed in B. The degraded type VI collagen detaches from the periphery of the trabecular beam (black arrow). [e: elastic-like tissue; G: ground substance].

As occurs in the CTM, the UTM core had an “empty” appearance in some regions due to the presence of abundant ground substance “Fig 6A”. In addition, there were elastic-like fibers and abundant type VI collagen, which intermingled with the collagen of the basal membranes (type IV) “Fig 6B”. In some zones the whole trabecular beam was filled up by collagen VI “Fig 6B”. The type VI collagen was degraded in the periphery of the trabecula and broken away from it “Fig 6B”. All endothelial cells lining the trabecular beams had disappeared due to cell death by necrosis “Fig 6”. In this surgical piece of trabeculectomy the ciliary muscle was not included.

#### Case 4

Patient 4 was a 8-year-old Caucasian girl who was diagnosed with PGC at birth. She underwent a goniotomy at HCSC 6 days after birth. A second and a third goniotomy performed at age 6 months and 16 months, respectively, kept IOP under control without medication until the age of 3 years. At that time, a trabeculectomy in the OD was performed because an increase in the papillary excavation was detected. At the age of 4, she underwent the same surgical technique applied to the OS due to poor IOP control. Since then, her IOP was controlled with three topical glaucoma medications in the OD and without medical treatment in the OS for a follow-up period of 4 years “Table 2”.

**Fig 7:**
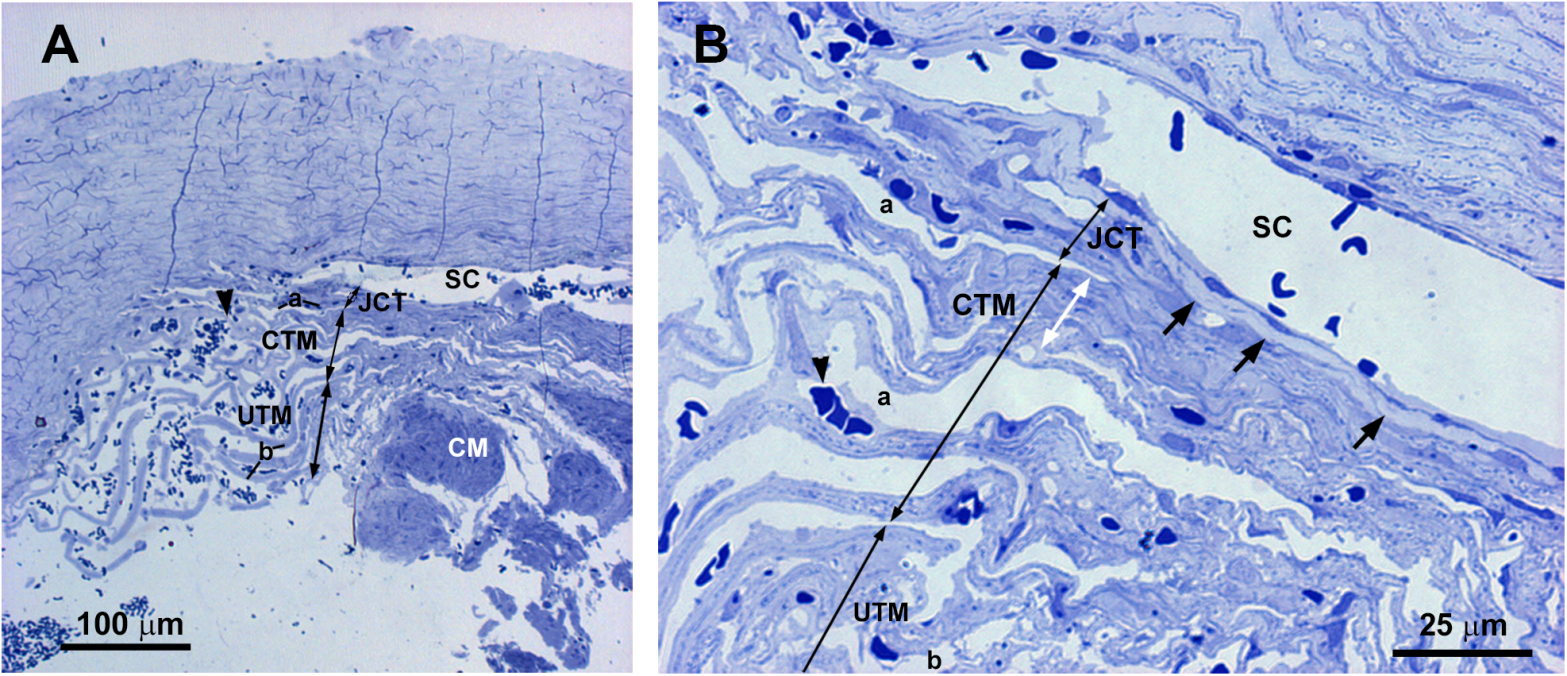
Case 4. **Trabeculectomy sample from the left eye.** Light micrographs. A: Schlemm’s canal (SC), juxtacanalicular tissue (JCT) (double arrows), corneoscleral trabecular meshwork (CTM) (double arrows) and uveoscleral trabecular meshwork (UTM) (double arrows) are shown. Intertrabecular spaces are evident in both the CTM (a) and UTM (b). The ciliary muscle (CM) is inserted overlapping the SC. Numerous red blood cells can be observed in all aqueous outflow pathways (arrowhead). B: High magnification of the image shown in A. The SC shows a large lumen. A well-defined light-blue band (arrow) is visible in the JCT (double arrows) next to the endothelial cells lining the inner wall of the SC. The CTM has evident intertrabecular spaces in some areas (a) while in others, the trabecular beams are fused (white double arrow).

**Fig 8:**
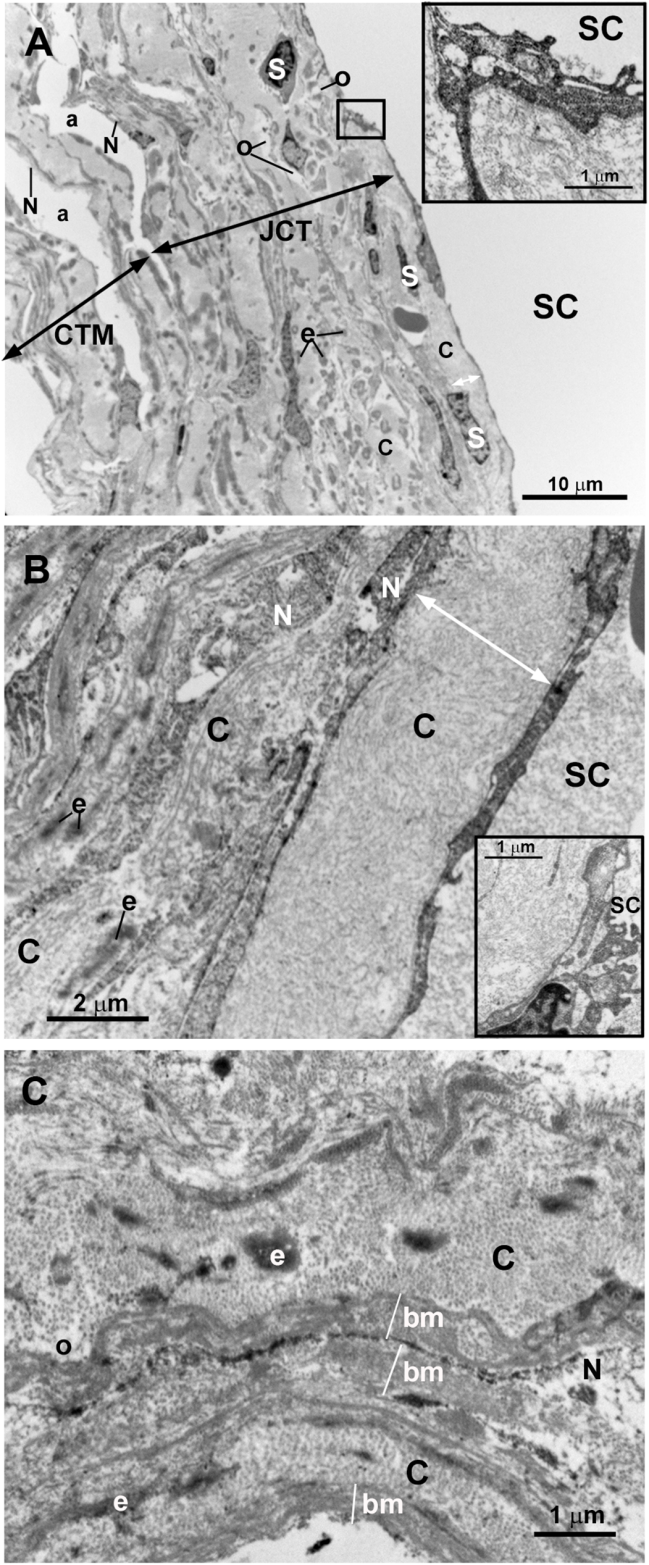
Case 4. **Trabeculectomy sample from the left eye.** Transmission electron microscopy. A and B: Schlemm’s canal (SC), juxtacanalicular tissue (JCT) (double arrows), and corneoscleral trabecular meshwork (CTM) (double arrows). Some endothelial cells lining the inner wall of SC are necrotic (inset in A) and others have some vacuoles (inset in B). The JCT has two differentiated areas: a band (double white arrow) formed mainly by coalescent fibrillary collagen (C) with scarce “optically empty spaces” (o). Next to this band the second area is composed of fibrillary collagen (C), abundant elastic-like fibers (e), stellate cells (some of them necrotic) (S), and “optically empty spaces” (o). Most endothelial trabecular cells are necrotic (N). C: High magnification of the corneoscleral trabecular meshwork beams. The trabecular core is filled with coalescent fibrillary collagen (C), elastic-like fibers (e) and few “optically empty spaces” (o). The trabecular beams are lined by necrotic endothelial cells (N) lying on a basal membrane (bm) which is thickened in some areas. [a: intertrabecular spaces in CTM].

**Fig 9:**
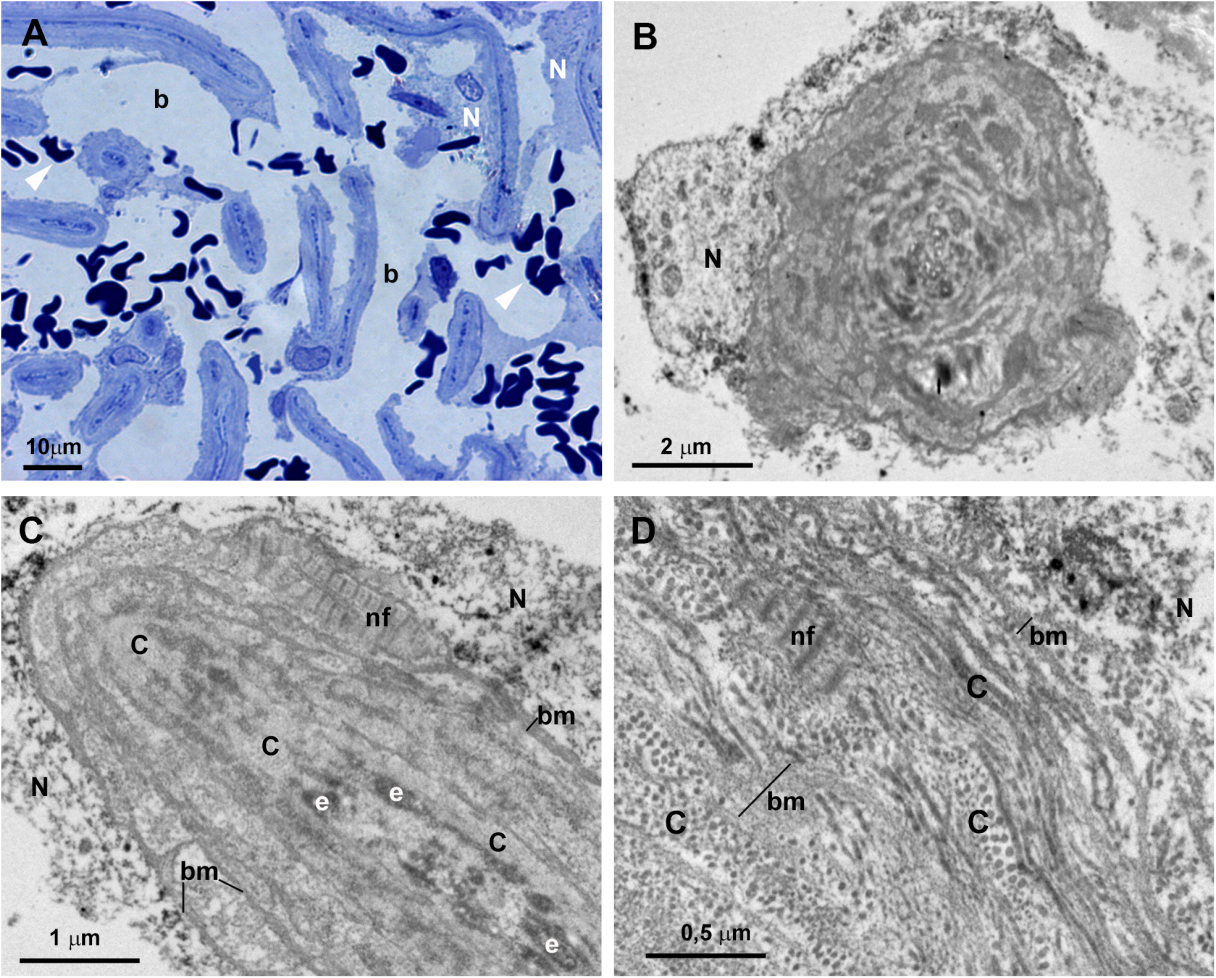
Case 4. **Trabeculectomy sample from the left eye.** A: Light micrographs. B-D: Transmission electron microscopy. A: Uveal trabecular meshwork. The intertrabecular spaces are open (b). Most of the trabecular endothelial cells have disappeared and those remaining are necrotic (N). Numerous red blood cells appear between the trabecular beams (arrowhead). B-D: High magnification of an uveal trabecular meshwork trabecular beam. B: Cross section. C and D: Longitudinal section. The trabecular core is filled with successive layers of collagen fibers (C) giving the appearance of onion-like layers. The collagen of the basal membrane (bm) is interspersed between successive layers of fibrillary collagen (C). In B-D necrotic remains of the endothelial cells lining the trabecular beams are observed (N). [e: elastic-like fibers; nf: non-fibrillary collagen VI].

In semithin sections obtained from the trabeculectomy of the OS, we observed a SC with a wide open lumen and a well-developed TM with abundant intertrabecular spaces “Fig 7”. No collector channels were observed “Fig 7A”. However, at TEM, most of the structures of the aqueous outflow pathways showed pathological changes. Some of the endothelial cells lining the inner wall of the SC were necrotic “Fig 8A inset” and in those remaining, few vacuoles were observed “Fig 8B inset”. The JCT had two clearly differentiated areas “Fig 8A and 8B”. The first one consisted of a thick band of fibrillary collagen densely packed “Fig 8B” located next to the endothelial cells of the inner wall of the SC. In this band, no JCT stellate cells were observed and the elastic-like fibers were difficult to find “Fig 8B”. In some zones, “optically empty spaces” “Fig 8A and 8B” were observed whereas in others the collagen fibers were so coalescent that those spaces could not be discerned “Fig 8A and 8B”. Underneath this band of collagen, a second area formed by stellate cells (some of them necrotic), abundant elastic-like fibers, fibrillary collagen and “optically empty spaces” was observed “Fig 8A”. In the CTM some of the trabecular beams were fused due to the disappearance of endothelial cells “Fig 8B and 8C” while others, showed large intertrabecular spaces “Fig 8A”. Most of the CTM trabecular endothelial cells were necrotic “Fig 8B and 8C”. The CTM trabecular core possessed abundant coalescent fibrillary collagen that mostly filled the trabecular beam. Besides, scarce “optically empty spaces”, elastic-like fibers and basal membranes (thickened in some areas), were visible “Fig 8C”. The UTM had large intertrabecular spaces “Fig 9A”. The core of the UTM beams was filled up by coalescent fibrillary collagen, basal membrane collagen and elastic-like fibers “Fig 9B–9D”. The collagen was arranged in onion-like layers “Fig 9B–9D”. Apparently, all the UTM endothelial trabecular cells were necrotic “Fig 9”. The ciliary muscle was abnormally inserted, overlapping the SC “Fig 7A”.

### Group C

#### Case 5

Patient 5 was a three-and-a-half-year old Caucasian female who was diagnosed with PGC at HCSC when she was one month old. This patient had an enlarged cornea, corneal edema and high IOP by digital palpation OU at presentation. A bilateral trabeculectomy was performed which resulted in corneal transparency and IOP control thereafter “Table 2”.

**Fig 10:**
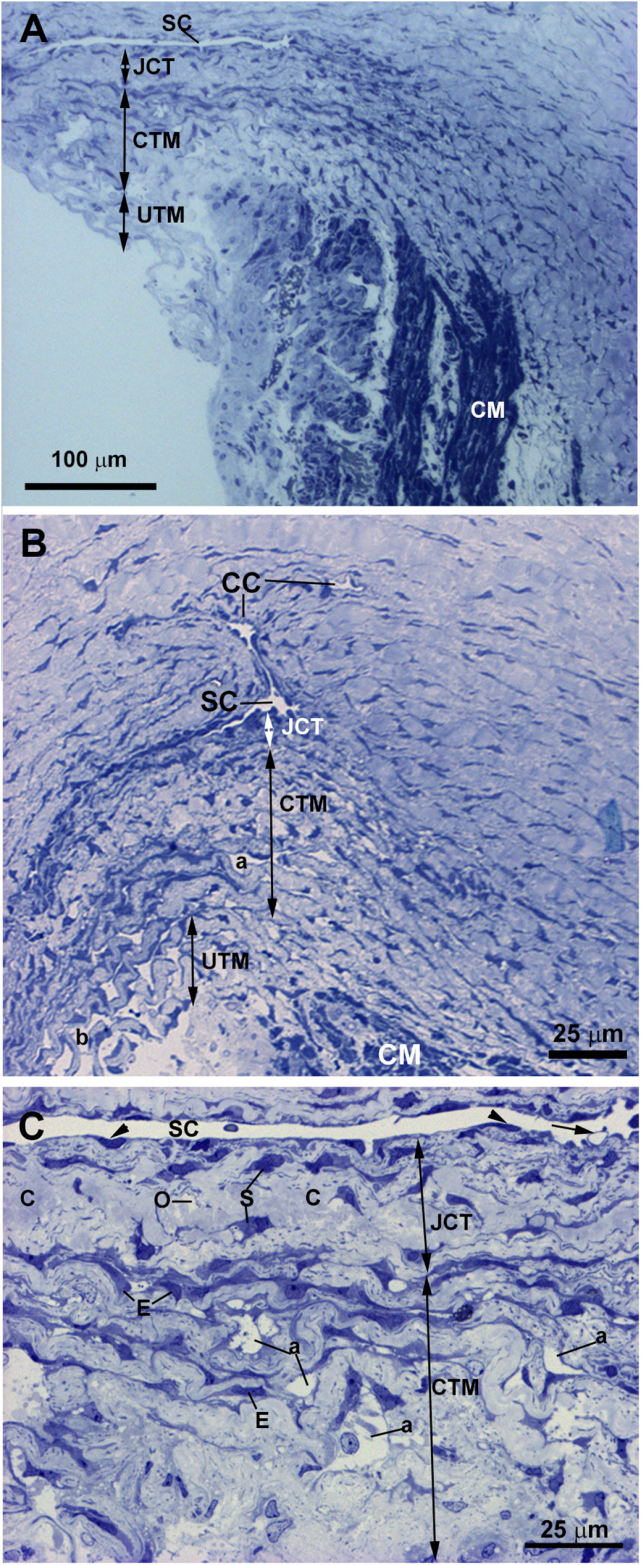
Case 5. **Trabeculectomy sample from the right eye.** Light micrographs. A: Schlemm’s canal (SC) with an open lumen is present. The ciliary muscle (CM) is inserted posterior to the SC. B: The SC and two collector channels (CC) are observed. The intertrabecular spaces of the corneoscleral (a) trabecular meshwork (CTM) (double arrows) are less evident than those of the uveal (b) trabecular meshwork (UTM) (double arrows). C: Endothelial cells lining the SC wall (arrowhead). The arrow points to a giant vacuole. The juxtacanalicular tissue (JCT) (double arrows) is thick and composed of stellate cells (S), collagen fibers (C), and “optically empty spaces” (o). The CTM has a compact appearance although intertrabecular spaces (a) are visible. [E: trabecular endothelial cell].

**Fig 11:**
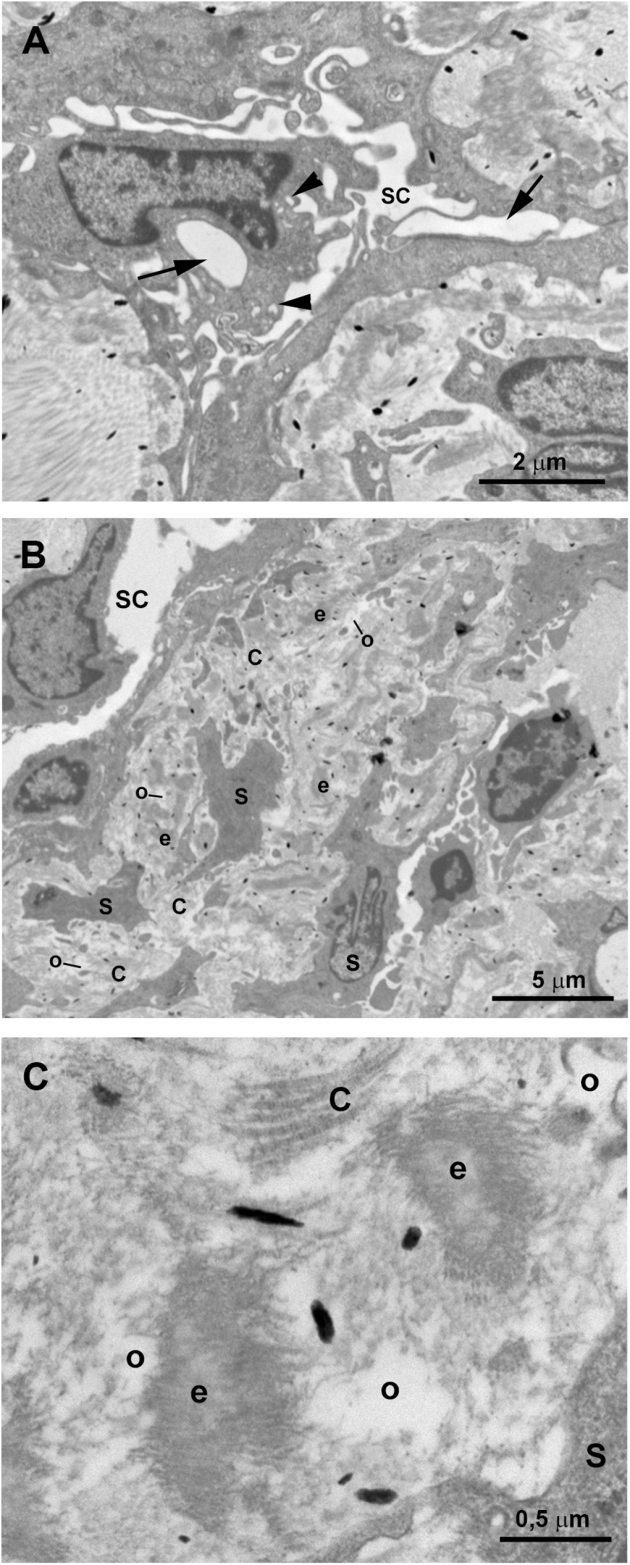
Case 5. **Trabeculectomy sample from the right eye.** Transmission electron microscopy. A: Schlemm’s canal (SC). The endothelial cells lining the inner wall of SC have some giant vacuoles (arrow) and numerous caveolae (arrowhead). B: Juxtacanalicular tissue (JCT). The thick JCT is constituted by disorganized fibrillary collagen (C), abundant stellate cells (S), elastic-like fibers (e) and “optically empty spaces” (o). C: High magnification of the JCT shown in B.

**Fig 12:**
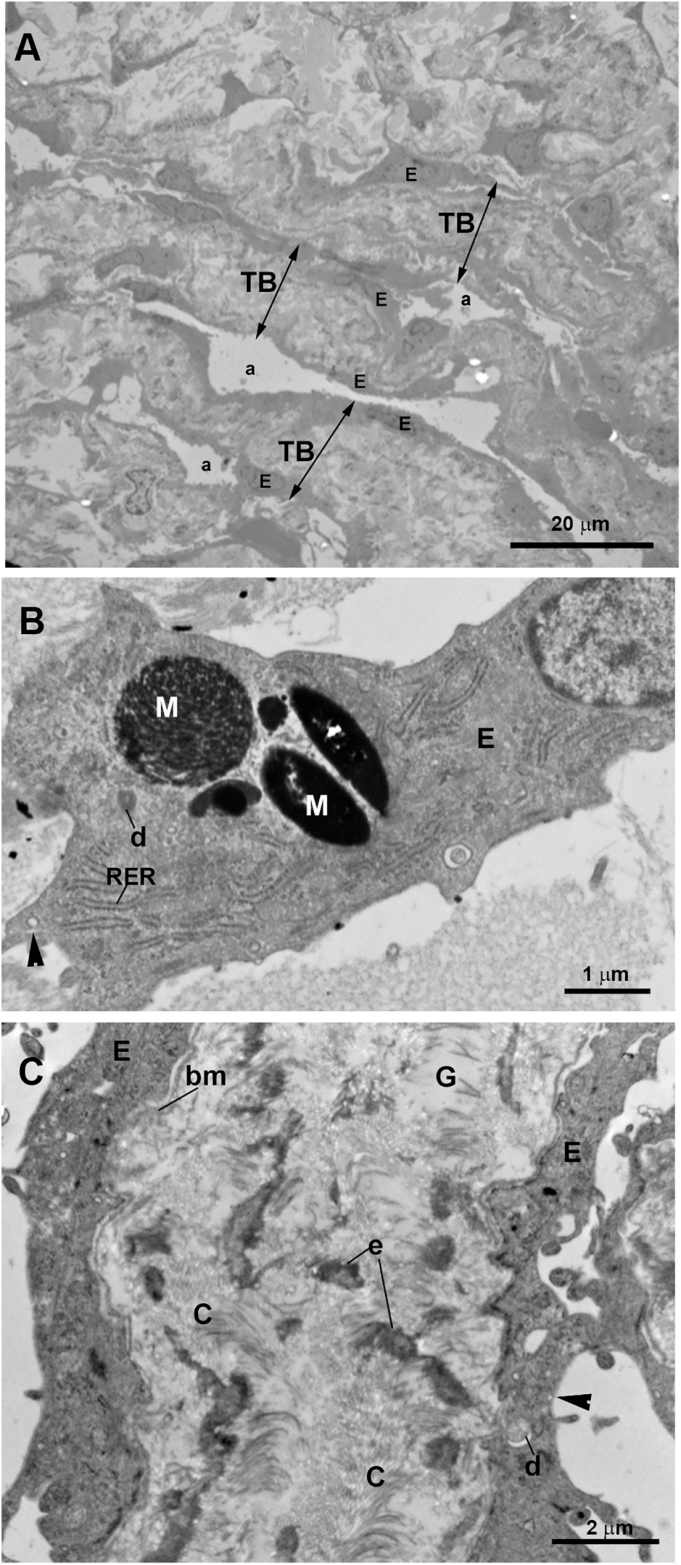
Case 5. **Trabeculectomy sample from the right eye.** Transmission electron microscopy. A: corneoscleral trabecular meshwork (CTM). This region is made up of several layers of trabecular beams (TB) (double arrows) separated by small intertrabecular spaces (a). Numerous flat endothelial cells (E) line the TB. B: High magnification of a trabecular endothelial cell (E). Pinocytosis vesicles (arrowhead), dense bodies (d), and phagocytized melanin granules (M) are visible within endothelial cells. Rough endoplasmic reticulum (RER). C: High magnification of a trabecular beam of the corneoscleral trabecular meshwork. The trabecular beams are filled by abundant disorganized fibrillary collagen (C), elastic-like fibers (e) and electron-lucent ground substance (G). The basal membrane (bm) of the trabeculae endothelial cells is observed. Pinocytosis vesicles (arrowhead).

**Fig 13:**
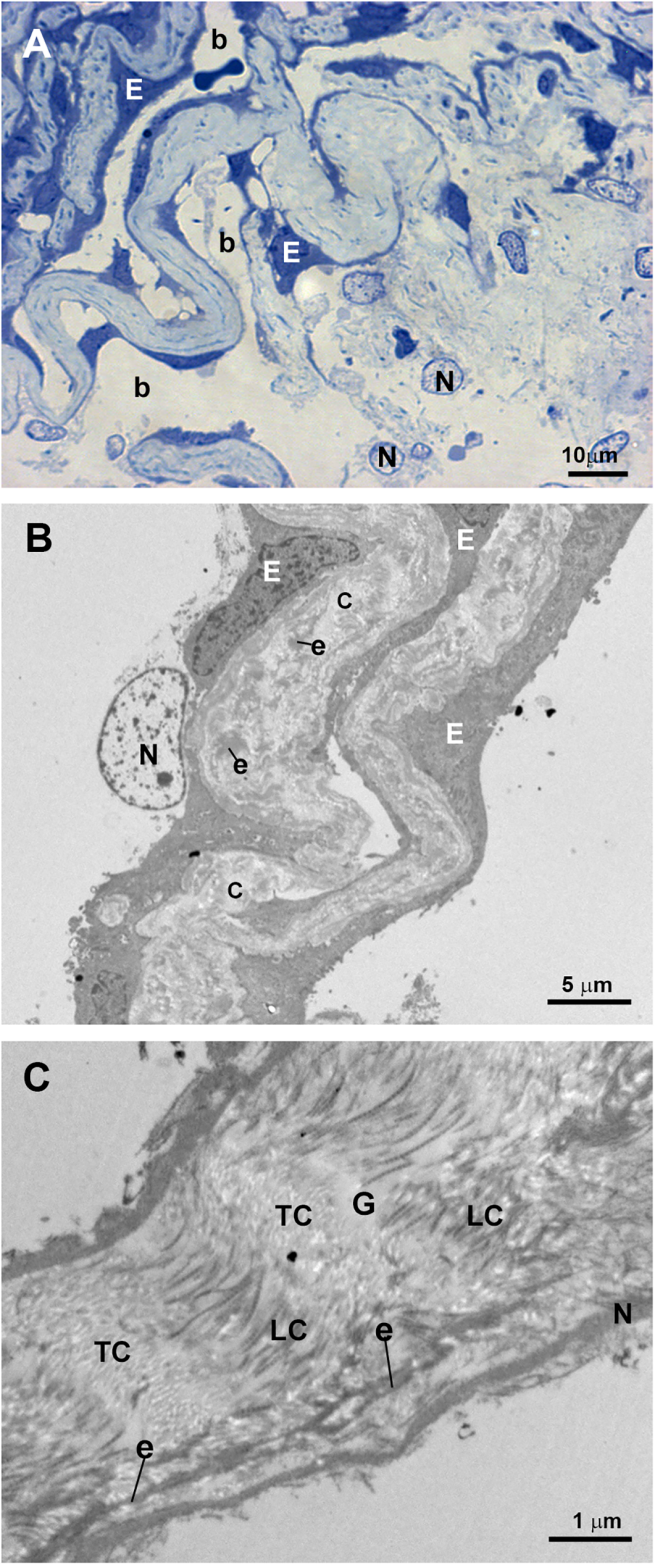
Case 5. **Trabeculectomy sample from the right eye.** A: Light micrograph. B and C: Transmission electron microscopy. A: The uveal trabecular meshwork is well differentiated. The intertrabecular spaces are open (b). Well-preserved endothelial cells (E) alternate with necrotic cells (N). B: Two uveal trabecular beams lined with well-preserved endothelial cells (E) and one necrotic cell (N). The trabecular beams are filled with abundant fibrillary collagen (C) and elastic-like fibers (e). C: High magnification of an uveal trabecular beam containing a large amount of disorganized coalescent fibrillary collagen. Cross-sectioned collagen fibers (TC) alternate with fibers sectioned longitudinally (LC). Scarce electron-lucent ground substance (G) between collagen fibers and elastic-like fibers (e) is visible. Debris of endothelial necrotic cells (N).

In this patient, the piece of trabeculectomy from the OD was considered for analysis. A developed SC “Figs 10, 11A and 11B” and collector channels “Fig 10B” with open lumens were observed. The endothelial cells covering de inner wall of the SC showed some giant vacuoles and abundant caveolae “Figs 10C and 11A”. The JCT was thick, due to the presence of abundant and disorganized fibrillary collagen and abundant stellate cells (resembling fibroblast) and elastic-like fibers “Figs 10 and 11”. “Optically empty spaces” were observed between the fibrillary collagen “Figs 10C, 11B and 11C”. All these findings result in a JCT with a compact appearance “Fig 11B and 11C”. The CTM was formed by several layers of trabecular beams. Although these trabeculae were quite compact, small intertrabecular spaces between them were visible “Figs 10B, 10C and 12A”. The trabecular beams were lined by endothelial cells which were separated from the core of the trabecula by a basal membrane “Fig 12C”. These cells showed numerous pinocytosis vesicles and dense bodies indicating a considerable phagocytic activity “Fig 12B and 12C”. The core of the CTM trabecular beam possessed abundant disorganized fibrillary collagen that mostly filled the entire trabecula “Fig 12C”. In addition, a few “optically empty spaces” containing an electron-lucent ground substance were observed “Fig 12C”. The UTM were more separate between them than the corneal ones “Figs 10A, 10B and 13A”. Many necrotic endothelial cells appeared “Fig 13A and 13B” together with a large amount of disorganized fibrillary collagen filling the trabecular core “Fig 13B and 13C”. Electron-lucent ground substance and elastic-like fibers were also found “Fig 13B and 13C”. The ciliary muscle was inserted posterior to the TM “Fig 10A and 10B”.

## Discussion

Although *CYP1B1* gene alterations have been identified as the main known genetic cause of congenital glaucoma, the underlying mechanisms remain poorly understood. Few previous reports on congenital glaucoma have described and debated microscopic alterations in congenital glaucoma structural terms, and only one previous attempt to correlate ultrastructural changes and genotype has been reported [22]. In this study, we have analyzed for the first time the *CYP1B1* genotype activity and the microscopic and clinical phenotypes in congenital glaucoma.

We found that patients predicted to have total absence of CYP1B1 enzymatic activity due to null genotypes presented variable goniodysgenesis. The degree of goniodysgenesis detected histologically was closely related to the severity of the disease and the difficulty for IOP control. The most severe goniodysgenesis corresponded to case 1 (group A) in which neither collectors channels nor SC were visualized, the TM was constituted by a compact tissue, and 7 surgical procedures were required for disease control. The next step of goniodysgenesis severity corresponded to group B (cases 2, 3, and 4) in which SC but no collector channels were observed and abnormal trabecular beams could be appreciated. This group of patients required between 2 to 4 surgical procedures to control the disorder. Both group A and B had an abnormal insertion of the ciliary muscle, which still overlapped the posterior portion of the TM, a position that would correspond to the end of fetal development (between 8 and 9 months of gestation) [25].

Notably, the mildest goniodysgenesis corresponded to the patient who was predicted to preserve approximately 60% or its CYP1B1 enzymatic activity (case 5, group C). In this case the collector channels and the SC were present and the TM was more developed than in group A and B. In addition, only one surgical procedure was needed for IOP control. However, the ciliary muscle in group C was also abnormally inserted although in a more posterior position than in groups A and B, evidencing that the repositioning of the ciliary muscle that normally takes place during angle development was absent. According to a typical recessive disease, this level of residual enzymatic activity would be expected to lead to a normal phenotype, which contrasts with the abnormal ultrastructural findings of this case. Therefore, one or more additional genetic alterations are likely to occur in patient 5, indicating the existence of modifier genes that modulate the phenotypic outcome. In this line, the observed intra- and interfamilial phenotypic variability associated with the same *CYP1B1* functional background also suggest the influence of modifier genes in the phenotype, supporting the contention that congenital glaucoma is not a simple genetic disease, according to previous observations [6, 19]. In support of the intrafamilial phenotypic variability associated with the same *CYP1B1* functional background are cases 2 and 3, two siblings that, despite sharing the same null *CYP1B1* genotype, presented differences: i) at time of disease presentation, at birth in case 2 and at age 5 in case 3; ii) in disease severity, case 2 requiring more surgical procedures and topical antiglaucoma medications than case 3 and; iii) in extracellular matrix composition of the outflow pathways, mostly coalescent collagen in case 2 vs. abundant ground substance in case 3. These ultrastructural differences could influence outflow facility and explain, at least partly, the disparity in clinical severity between siblings.

According to our results, a previous report described an association of severe or moderate angle abnormalities with specific CYP1B1 mutations in congenital glaucoma patients. However, data concerning the genotype-associated enzymatic activity were not included. Nevertheless, it can be predicted that one of the patients described in that study (patient 3), who presented moderate goniodysgenesis, carried a CYP1B1 genotype (p.Arg368His/p.Gly61Glu) inferred to be associated with very low enzymatic activity based on the functional features of these mutations [18, 26]. No mention to the collector-channel development is found in the work of Hollander et al. [22]; however, given that in the microphotograph of Hollander’s patient 3 the SC is present but no collector channels are visible, we hypothesized that the degree of angle development of this case would correspond to our group B, in accordance with our prediction of low CYP1B1 enzymatic activity.

In addition, three out of a total of six patients of the Hollander’s study were compound heterozygotes for *CYP1B1* rare variants, and one of them (patient 5) was a single heterozygote for the hypomorphic p.Glu229Lys variant, which retains about 40% activity of the wild-type [19]. Therefore, the estimated enzymatic activity associated with this genotype is approximately 70%. This patient presented a moderate goniodysgenesis in Hollander’s classification [22]. Given that only data about SC is included in Hollander’s patient 5, whether this goniodysgenesis would correspond with our group B or C could not be deduced.

Apart from the observation of SC and collector channels, in the present study other ultrastructural characteristic of the outflow pathway which could be related to CYP1B1 enzymatic activity were analyzed. Among them: i) necrosis of the endothelial cells lining the trabecular beams; ii) variable underdevelopment of outflow pathways; and iii) increased ECM in the TM tissue.

The endothelial trabecular cells have important roles in TM function: a) regulating the balance between ECM components (collagen, elastin, proteoglycans, etc.) secretion and their degradation by metalloproteinases [27]; b) acting as phagocytes in removing cell debris, thus preventing blockage of the TM [28]; c) modulating outflow facility by a dual mechanism, their contractile capacity [29] and changes in cell volume [30]. Therefore, endothelial trabecular cells damage or loss could be involved in IOP raise. It has been demonstrated that mice with null *CYP1B1* genotype *(Cyp1b1*^*-/-*^*)* show impaired trabecular cell function and oxidative homeostasis [31]. *Cyp1b1*^*-/-*^ TM cells presented an insufficient cellular antioxidant capacity to detoxify reactive oxygen species (ROS), a deficiency that impairs growth, development, and differentiation of TM tissue [31]. It has been postulated that the increased oxidative stress noted in these animals could produce DNA damage in the cellular components of the outflow pathways, which could result in reduced TM cell adhesion and death, situations that could compromise TM integrity and result in pathologic changes [32, 33]. TM tissues from *Cyp1b1*^*-/-*^ mice [34] and cultured TM cells from the same animal model [31] presented increased cell-death rates. According to these data, we observed that most of the trabecular endothelial cells from patients predicted to carry null *CYP1B1* genotypes (groups A and B) were necrotic. In the study by Hollander et al (2006) [22] the patient that carried a *CYP1B1* genotype (p.Arg368His/p.Gly61Glu), predicted to be associated with very low enzymatic activity, could correspond with our group B, as mentioned above. Similarly to our group B of patients, Hollander’s case had denuded corneoscleral trabeculae. By contrast, in our group-C patient, which was predicted to preserve approximately 60% or its CYP1B1 enzymatic activity, most of the trabecular endothelial cells were still alive with abundant pinocytosis vesicles and dense bodies, indicating a considerable phagocytic activity. In this patient, necrotic cells were detected only in the UTM. A possible explanation for this finding could be the higher mechanical stress induced by the IOP in this area. In view of the aforementioned findings, it seems that null *CYP1B1* genotypes, associated with very low enzymatic activity, could be associated with trabecular endothelial cell death.

As in the most severe dysgenesis cases described by Hollander (2006) [22] we did not find the SC in the patient of group A. The TM findings in our study ranged from: i) a TM comprised by a compact tissue (group A), ii) a CTM consisting in fused and denuded trabecular beams (group B); and iii) a CTM formed by dense trabecular beams and small intertrabecular spaces (group C). These findings are consistent with those reported in mice deficient in *CYP1B1 (Cyp1b1*^*-/-*^*)*, which showed angle abnormalities such as small or absent SC and hypoplastic TM that, on occasions, can be completely collapsed [31, 34–36]. In addition, in the present study, we did not observed collector channels in the patients of groups A and B, both having a total absence of CYP1B1 enzymatic activity due to null genotypes. During normal human angle development, the SC and the anlage of the collector channels sprouting from its outer wall are observed at 24 weeks of gestation; then the primordium of the intrascleral plexus is present at 36 weeks of gestation [37]. From these findings it can be deduced that the post-trabecular structures develop in direction of the sclera. Therefore, the fact that we did not observed collector channels in some of our samples could be attributed to arrested growth and development of angle structures.

The cellular component of outflow pathways share many characteristics of vascular endothelial cells [38]. An important role for the expression of *CYP1B1* in vascular development has been suggested [39–42]. Vascular endothelial cells of *Cyp1b1*^*-/-*^ mice failed to undergo capillary morphogenesis [43]. This fact could explain, at least in part, the underdevelopment of the collector channels and SC observed in patients with mutations in *CYP1B1*.

In our study, all patients showed an increment in the ECM of the TM and JCT. However, given that in the present work the two patients predicted to have total absence of CYP1B1 enzymatic activity and the patient predicted to preserve approximately 60% or its CYP1B enzymatic activity, showed an increase in the ECM in the trabecular beams and in the JCT, it seems reasonable to postulate that the increase in ECM could not be directly related to the *CYP1B1* mutation. The mechanical stress, induced by increase in chronic IOP secondary to outflow pathway dysgenesis, could be responsible for the increase and disorganization of the ECM components, especially collagen, observed in all the patients of our study as well as in those reported by Hollander (2006) [22]. All these ECM changes in the TM result in stronger resistance of aqueous humor outflow and thus could influence the maintenance of an elevated IOP.

A common limitation of these types of studies is the difficulty to obtain large numbers of tissue samples from patients carrying known mutations. Even to get short series of samples, several years of follow-up are usually required. In addition, is also difficult to have access to child cadaver donors to be used as controls due to the low birth rate and infant-mortality rate in Spain. In addition, technical issues of working with human samples of trabeculectomy have been reported. Among these are the small size of the surgical tissue available and the difficulty in orienting the specimens, namely to position the samples with the trabecular surface upwards and to determine the anteroposterior orientation of the surgical pieces [44].

The most relevant histological findings in the outflow pathway in our patients with PCG and mutations in *CYP1B1* were: i) underdeveloped collector channels and the SC; ii) abnormal insertion of the ciliary muscle and; iii) death of the trabecular endothelial cells. Our observations could be useful in improving the treatment strategy of PCG associated with mutations in *CYP1B1*. Thus, for a patient with a total absence of CYP1B1 enzymatic activity due to null genotypes, the clinician should take into account the possibility of an underdeveloped post-trabecular outflow pathway (SC and/or collector channels) and therefore, advise a filtration procedure or a pediatric valve at the outset of the disease instead of beginning with a non-filtering procedure that most probably would fail to control IOP.

In summary, our results further support the role of *CYP1B1* gene dysfunction on goniodysgenesis. Although overall goniodysgenes and clinical features associated with *CYP1B1* mutations are severe, there is some degree of variability, which suggests the role of modifier factors. This is not surprising if we consider that anterior segment chamber development is a complex process controlled by numerous genes, whose alleles may differ in their corresponding biological activities. Future global analysis of the genome variants in these patients may help to unravel the underlying pathogenic mechanisms in congenital glaucoma.
